# Composition, Structure, and PGPR Traits of the Rhizospheric Bacterial Communities Associated With Wild and Cultivated *Echinocactus platyacanthus* and *Neobuxbaumia polylopha*

**DOI:** 10.3389/fmicb.2020.01424

**Published:** 2020-06-26

**Authors:** María Eugenia de la Torre-Hernández, Leilani I. Salinas-Virgen, J. Félix Aguirre-Garrido, Antonio J. Fernández-González, Francisco Martínez-Abarca, Daniel Montiel-Lugo, Hugo C. Ramírez-Saad

**Affiliations:** ^1^CONACYT-Universidad Autónoma Metropolitana-Xochimilco, Mexico City, Mexico; ^2^Maestría en Ciencias Agropecuarias, Universidad Autónoma Metropolitana-Xochimilco, Mexico City, Mexico; ^3^Departamento de Ciencias Ambientales, Universidad Autónoma Metropolitana-Lerma, Estado de México Mexico; ^4^Grupo de Ecología Genética, Estación Experimental del Zaidín, Consejo Superior de Investigaciones Científicas, Granada, Spain; ^5^Departamento Sistemas Biológicos, Universidad Autónoma Metropolitana-Xochimilco, Mexico City, Mexico

**Keywords:** *Echinocactus platyacanthus*, *Neobuxbaumia polylopha*, semiarid, bacterial-community, rhizosphere, PGPR

## Abstract

The Queretaro semi-desert in central Mexico is the most southern extension of the Chihuahua desert. This semi-arid zone shelters a vast cactus diversity with many endemic species. Currently, two cacti species from this semi-desert namely, *Echinocactus platyacanthus* and *Neobuxbaumia polylopha* are under a threat to their survival. So far, there are no reports on the bacterial communities associated with these plants. In this study, we assessed the structure and diversity of the rhizospheric bacterial communities associated with *Echinocactus platyacanthus* and *Neobuxbaumia polylopha* growing in wild and cultivated conditions. Samples of *E. platyacanthus* were also approached with culture-based methods in search of isolates with plant growth promoting abilities. Metagenomic DNA was extracted from rhizospheric samples and used for Illumina sequencing of the 16S rRNA gene. α-diversity and amplicon sequence variant (ASV) richness were higher in both groups of *E. platyacanthus* samples. All samples accounted for 14 phyla, and the major 6 were common to all treatments. The dominant phyla in all four sample groups were *Actinobacteria* and *Proteobacteria*. Analysis at family and genus levels showed association patterns with the cultivated samples from both species grouping together, while the wild samples of each cactus species were grouping apart. High abundance values of Rubrobacteraceae (15.9–18.4%) were a characteristic feature of wild *E. platyacanthus* samples. In total, 2,227 ASVs were scored in all 12 rhizospheric samples where *E. platyacanthus* samples showed higher richness with 1,536 ASVs. Regarding the growing conditions, both groups of cultivated samples were also richer accounting for 743 and 615 ASVs for *E. platyacanthus* and *N. polylopha*, respectively. The isolates from *E. platyacanthus* rhizosphere were mainly assigned to *Bacilli* and *Gammaproteobacteria*. In total 35 strains were assayed for PGPR traits (IAA and siderophore production, phosphate solubilization, and fungal growth inhibition). Strains obtained from plants growing in the wild displayed better PGPR characteristics, stressing that naturally occurring wild plants are a source of bacteria with diverse metabolic activities, which can be very important players in the adaptation of cacti to their natural environments.

## Introduction

Arid areas are characterized by poor water supply, as their precipitation and atmospheric humidity are below the annual global average. In arid areas annual rainfall account for 250 mm or less, while in semi-arid zones the record of precipitation is between 250 and 500 mm per year (González-Medrano, 2012). The extension of arid and semi-arid zones (ASZ) in the world is constantly increasing, due to a process called desertification (Muñoz-Meléndez, 2019). This process becomes irreversible when the primary vegetation disappears, followed by the loss of soil, and finally emerging the bare rock, which lacks water deposits necessary for the establishment and survival of plants (González-Medrano, 2012; Granados-Sánchez et al., 2013; Landa et al., 2014).

In Mexico, the area of the ASZ has increased markedly in the last 25 years. Since 1994, it comprised 41% and currently represents 60% of the national territory (CONAZA, 2019). Despite the extreme conditions of ASZ, a large number of plant species, mostly cacti are found in these regions (Hernández-Oria et al., 2007; Hernández-Magaña et al., 2012). The largest and most important arid region in Mexico is the Chihuahua desert, and at its southern end is the Queretaro semi-desert that comprises the Tolimán quadrant, which shelters the largest cactological wealth of the Chihuahua desert (Hernández et al., 2007; Hernández-Oria et al., 2007). The quadrant comprises of 55 registered cacti species (Hernández-Magaña et al., 2012) 13 of which are endemic and 17 are included in some threatening category according to the national normative NOM – 059 (SEMARNAT 2010).

The *Cactaceae* family is native to the American continent comprising about 2000 species (Hernández et al., 2007; Jiménez-Sierra, 2011; Castañeda-Romero et al., 2016). Members of this plant family have successfully colonized arid and semi-arid environments, in the herbaceous, shrubby and arboreal strata, becoming key elements in the structure and dynamics of the plant communities of the ASZ. Different studies have addressed the ecological importance of cacti in ASZ (Valiente-Banuet et al., 1991; Arriaga et al., 1993; Le Houérou, 1996; Bashan et al., 2000; Carrillo-Garcia et al., 2000a; Jiménez-Sierra, 2011). Nevertheless, their characteristic slow development and the poor survival of young individuals, makes them especially vulnerable to the decline of their populations (Meza-Nivón, 2011; Hernández et al., 2013). Besides, cacti also have great socio-economic value, as they are intensively extracted for various purposes such as ornamental, crafts, construction, human and animal food (Ramírez, 2005; Jiménez-Sierra and Eguiarte, 2010; Granados-Sánchez et al., 2013; Royo-Márquez et al., 2014).

*Cactaceae* like most plants benefit from soil microorganisms being dependent on their presence, and association for their early establishment and subsequent development (de-Bashan et al., 2007). The study of the microbial communities associated with different cacti species has been explored. There is a group of different bacterial species capable of promoting plant growth (PGPR). They are mainly associated with the rhizosphere of plants, where they develop various activities that help to increase plant growth and productivity (de-Bashan et al., 2007; Verma et al., 2010). Inoculation with PGPR as bio fertilizers or pest control agents is a common practice in agriculture and forestry in developed countries (Rodrìguez and Fraga, 1999; Bashan et al., 2005), however, its use is still limited in developing countries (Bashan, 1998).

In Mexico, initial studies with PGPR and cacti-associated bacteria addressed the effect of inoculating *Azospirillum brasilense* on the giant cardon (*Pachycereus pringlei*). Puente and Bashan (1993) reported higher seed germination, and development of inoculated cacti after 9 months, while Carrillo-Garcia et al. (2000b) found improved root and shoot growth of inoculated cardon plantlets growing in poor soils. *A. brasilense* has also been used to inoculate; *Pachycereus pringlei, Stenocereus thurberi*, and *Lophocereus schottii*, in the nursery and later transplanting them in order to stabilize eroded soils in the Baja California desert. This resulted in higher survival and development rates as compared with non-inoculated control plants. In addition, soil erosion diminished in the experimental plots containing inoculated plants (Bashan et al., 1999). Different bacterial strains isolated from the rhizosphere of *Coryphantha radians* and *Mammillaria magnimamma* were used to promote the growth of *Mammilaria zeilmanniana* (Chávez-Ambriz et al., 2016). Other studies have analyzed the diversity of rhizospheric bacterial communities associated with *Stenocereus stellatus, Mammillaria carnea*, and *Opuntia pilifera*, endemic cacti of the Tehuacán-Cuicatlán Biosphere Reserve (Aguirre-Garrido et al., 2012; Torres-Cortés et al., 2012). Fonseca-García et al. (2016) addressed the structure of archeal, bacterial and fungal communities associated with *Myrtillocactus geometrizans* and *Opuntia robusta*, in five plant compartments and bulk soil. Recently, the effect of soil contamination by Zn on the diversity of the microbiota associated with *Echinocactus platyacanthus* was analyzed to explain its high tolerance to environmental stress (Sarria-Carabali et al., 2019). While in Brazil Lima et al. (2015) characterized endophytic bacteria isolated from *Cereus jamacaru* and *Melocactus zehntneri*. Kavamura et al. (2013) focused on the cultivable fraction of drought-tolerant bacteria associated with cacti from the arid Caatinga region. However, there are several cacti species whose associated microbiota remains unknown.

*Echinocactus platyacanthus* and *Neobuxbaumia polylopha* are two cacti species present in the Queretaro semi-desert. Currently, their natural populations are under a threat to their survival (IUCN, 2019) besides the recent report by Sarria-Carabali et al. (2019) there are no further studies on the bacterial communities associated with the rhizospheres of these cacti. The candy barrel cactus or sweet biznaga (*E. platyacanthus*), is a globular cactus that measures up to 1.5 m high by 1 m wide (Arias-Montes et al., 2012). *E. platyancanthus* used to be a relatively abundant species, however, due to a high demand of young specimens used for the preparation of acitron (a traditional candy), their numbers have been vastly reduced. The golden organ (*N. polylopha*), is a columnar cactus characterized by the golden coloration of its crown and spines, becoming highly valued as an ornamental plant. Natural populations of *E. platyacanthus* and *N. polylopha* can still be found in several wild spots of the Queretaro semi-desert, although they do not develop in vicinity as their growing conditions differ. The former species prefers ravines with calcareous soils, while the latter is generally found in hillsides with slight slopes and plateaus with stony soils, poor in organic matter (Eguiarte and Jiménez-Sierra, 2000). In the urban and suburban areas of the Queretaro semi-desert, few cultivated individuals of both plant species can also be found in some public and private gardens, but mainly in nurseries where they have been successfully propagated under controlled conditions. However, these cultivated plants are not suitable for reintroduction in wild areas as their survival rate is very low or null.

This study aimed to characterize under an NGS approach, the rhizospheric bacterial communities associated with *E. platyacanthus* and *N. polylopha* growing in wild and cultivated conditions. Samples of *E. platyacanthus* were also assessed under a culture-based approach. Isolated rhizospheric bacteria were further identified and characterized in search of plant growth promoting abilities.

## Materials and Methods

### Sampling Sites and Samples Collection

Wild samples of *N. polylopha* (NpW) were collected from a natural stand growing at Huajales, Queretaro State (Qro). This area is located at the border between deciduous forest and oak forest; the soils are shallow, calcareous, and poorly developed, classified as Regosols and Pheosem. Wild samples of *E. platyacanthus* (EpW) were taken from a natural population growing in the outskirts of Toliman town, Qro. The vegetation are crassicaullous and submontanous scrubs, the soils are in general shallow, stony, corresponding to calcareous Leptosols. Samples of cultivated *N. polylopha* specimens (NpC) were obtained from two different nurseries in the town of Cadereyta, Qro (10 km S of Toliman), and those from cultivated *E. platyacanthus* plants (EpC) were obtained from specimens growing in gardens of two public offices in Toliman town. All samples were collected at the end of the 5 month rainy season (late October). Samples of secondary roots and adhering soil were taken from both cacti species, in each growing condition (wild and cultivated). Typically, 0.75–2 g were obtained depending on plant size, trying to have plants of different sizes in each group of samples. Plant sizes for Np plants were considered as follows: sample 1, plants with height between 25 and 100 cm; sample 2, plants between 1 and 5 m height; sample 3, plants higher than 5 m. For Ep samples, the range of sizes was sample 1, plants with diameter between 5 and 25 cm; sample 2, plants between 25 and 50 cm diameter; sample 3, plants with more than 50 cm diameter. According to these features, the 12 samples obtained were identified as *N. polylopha* cultivated NpC-1, NpC-2, NpC-3; *N. polylopha* wild: NpW-1, NpW-2, NpW-3; *E. platyacanthus* cultivated: EpC-1, EpC-2, EpC-3; *E. platyacanthus* wild: EpW-1, EpW-2, EpW-3. Samples were put in self-sealing plastic bags and transported in an insulated container to the lab. Processing of samples was done within the next 2 days. Soil characteristics and biogeographic data of the sampling sites are provided in Table 1.

**TABLE 1:** Geographical location, climate and physicochemical characteristics of the soil from the sampling areas. Geographical data for Nurseries, Tolimán and Garden are the same as they are close localities.

		Huajales			Nurseries		Toliman gardens			
		NpW1	NpW2	NpW3	NpC1-2	NpC3	EpW1	EpW2	EpW3	EpC2-3
Altitude (meters above sea level)^a^		1080			1560					
Coordinates		21°12′36″N/99°31′6.4″W			20°55′1.1″N/99°56’15.5″W					
Weather		Warm sub humid			Dry semi-warm					
Annual precipitation (mm)^a^		952			406					
Annual mean temperature (°C)^a^		19.8			15.3					
Soil type^b^		Regosols and Pheosem					Leptosols			
**Soil physicochemical characteristics**										
Density (g/cm^3^)		0.9	1	0.8	1.6	1.5	1.6	1.7	1.4	1.6
Total porosity (%)		51.1	52	56.3	24.2	25.2	33.3	34.7	32.4	39.5
Texture	Sands (%)	95.4	95.3	95.8	92	95.4	90.2	90.3	90.4	77.8
	Silts (%)	1	1.6	1.3	1.3	1.4	0.5	8.3	0.5	21.9
	Clays (%)	3.6	3.1	2.9	6.7	3.2	9.3	1.4	9.1	0.2
	Textural classification	S	S	S	S	S	S	S	S	SL
pH in H_2_O (1:2.5)		7.35	7.6	6.64	7.78	8.16	8.13	8.05	8.07	7.29
Organic matter (%)		6.6	6.48	6.6	2.91	2.5	2.5	2.91	4.22	1.9
Organic C (%)		3.83	3.76	3.83	1.69	1.45	1.45	1.69	2.45	1.1
	Calcium (Ca^2+^)	36	20	29	11	8	36	13	33	nd
Exchangeable cations	Magnesium (Mg^2+^)	31.5	19	14.25	1.5	50.5	31.5	64.5	27	nd
Meq-100g soil	Sodium (Na^–^)	3.26	2.83	3.26	3.91	5	2.39	2.5	2.39	nd
	Potassium (K^+^)	1.54	1.54	1.15	2.44	2.95	0.9	1.22	1.54	nd

*^*a*^Data from: http://smn.conagua.gob.mx; ^*b*^data from: http://inegi.org.mx/temas/edafologia; S = Sandy, SL = Sandy-Loam; nd = not determined.*

### DNA Extraction, PCR Amplification, and Massive Sequencing

Rhizospheric soil from each sample was collected by gently brushing the soil adhering to the roots. Aliquots (± 0.25 g) from these rhizospheric soils were used to extract metagenomic DNA with the PowerSoil DNA isolation kit (cat. number: 12888-100), following manufacturer’s instructions. The integrity of extracted DNA was checked by electrophoresis on 1% agarose gel, stained with ethidium bromide (0.5 μg mL^–1^). Amplificability of obtained DNA was checked in the lab on reactions targeting the V6–V8 region of the 16S rRNA gene. After standardizing DNA concentrations, aliquots were sent to the sequencing service of Integrated Microbiome Resource, at Dalhousie University, Canada, to be processed in an Illumina MiSeq platform.

### Illumina Data Processing

Raw reads were processed with DADA2 (Callahan et al., 2016) an open-source package running under R environment. The pipeline tutorial in the website^1^ was followed. In the filtering and trimming steps, the R1 reads from all the samples in the dataset were trimmed by keeping the first 275 nt, and for the R2 dataset only the first 250 nt. Then, R1 and R2 reads were merged using default parameters. In the initially obtained table (seqtab) of amplicon sequence variant (ASV), only reads between 383 and 411 nt were further used. Chimeras were detected and discarded with “removeBimeraDenovo” command. The remaining ASVs were classified with an 80% bootstrap cut off, by comparing against the 16S rRNA reference database of the Ribosomal Database Project II, training set v.16 (Cole et al., 2014) and the assign Taxonomy command from DADA2 was used for this purpose. The resulting dataset was further run with the classify.seqs command from MOTHUR v.1.42.1 (Schloss et al., 2009). This MOTHUR command was used due to the lack of ability of the former command to assign mitochondrial sequences in the datasets. This problem is also related to the RDP database structure; however, in the last version of MOTHUR (trainset16_022016.tax), the RDP taxonomy file was formatted to avoid the problem. Finally, in this refined mitochondria-free dataset, ASVs accounting for less than 0.005% of the total sequences in the dataset were removed according to Bokulich et al. (2013).

### Statistical Analyses

Alpha diversity indices and other community parameters (Observed ASV and Chao1 for richness; Shannon, Simpson, InvSimpson and Evenness indices) were obtained with the tool set Phyloseq (McMurdie and Holmes, 2013) and compared using one-way ANOVA test, followed by Tukey HSD as *post-hoc* test. These tools and tests were run under R package. Normality and homoscedasticity of the alpha parameters were checked with shapiro.test and levene.test commands, respectively. For the alpha diversity analyses, filtered ASV sequences were randomly subsampled (rarefied) to 5213 reads per sample. For the beta diversity analyses, ASV counts were normalized by using the “trimmed means of M” (TMM) method with the BioConductor package edgeR (Robinson et al., 2010). ASV sequences were aligned with MAFFT online version^2^ with default parameters, and a tree was constructed with FastTree v.2.1.11, using the GTR method and Gamma20 likelihood optimization (Price et al., 2010). The resulting tree was used to calculate Weighted UniFrac Distances. These normalized distance values were considered to perform the permutational analysis of variance (PERMANOVA), and permutational analysis of multivariate homogeneity of group’s dispersions (BETADISPER), with 9,999 permutations. For these analyses, the respective functions adonis and betadisper in the R vegan package were used (Oksanen et al., 2019). PERMANOVA test was performed comparing wild vs. cultivated plants in each plant species, and fixing the species with the strata parameter in the adonis command. To visualize the similarities or dissimilarities of the studied communities, a Principal Coordinates Analysis (PCoA) was plotted by using Weighted UniFrac distances to ordinate in two dimensions the variance of beta diversity among all treatments. Ordination analyses were performed using the R package Phyloseq and the above-mentioned tree. The relative abundance values from phyla, classes, orders, families, and genera were assessed with ANOVA, and Games-Howell’s *post hoc* test, by applying Benjamini-Hochberg FDR correction for multiple tests, using STAMP v.2.1.3 software (Parks et al., 2014). Venn diagrams were constructed with the web tool InteractiVenn^3^.

### Microbiological Approach

#### CFU Counts and Isolation

Equal-size aliquots from each rhizospheric soil were pooled together according to their treatment. Suspensions (1:10) of these four pooled samples were followed by 8 successive decimal dilutions of each one. Aliquots of these dilutions were inoculated on TY agar plates, incubated, and used for viable counts according to Bonilla et al. (2016). After colony scoring, the plates were also used to obtain a collection of rhizospheric bacterial isolates, by selecting single colonies with different morphology.

#### Extraction of Genomic DNA and 16S rRNA Gene Amplification From Isolates

Genomic DNA from all rhizospheric isolates was extracted with the Roche High Pure PCR Template Preparation Kit (Cat. # 11 796 828 001), according to the manufacturer’s instructions. The 16S rRNA gene was amplified by PCR with primers 8F (Turner et al., 1999) and 1492R (Weisburg et al., 1991). The obtained products were purified with Promega’s Wizard SV Gel and PCR Clean-Up System kit (Cat. A9281). The size and integrity of genomic DNA and PCR products were evaluated in 1% agarose gels stained with ethidium bromide (0.5 μg mL^–1^).

### Grouping of Isolates by ARDRA

Grouping of the collection of rhizospheric isolates was done by Amplified Ribosomal DNA Restriction Analysis (ARDRA) of nearly full-length amplicons of the 16S rRNA gene (Massol-Deya et al., 1995). The enzymes *Hae*III (GG/CC) and *Msp*I (C/CGG) were used in a single reaction (Jose and Jebakumar, 2012). The restriction patterns were observed in 2% agarose gels and documented in an EDAS 290 System. Clustering of the restriction patterns was made with the 1D Image Analysis Software (Kodak, Inc.).

### Phylogenetic Analysis of Bacterial Isolates

Representatives of the obtained ribotypes were subjected to 16S-based phylogenetic analysis. Amplicons of nearly full-length 16S rRNA gene (∼1,450 nt) from the bacteria representing the obtained ribotypes were sequenced by external services (Macrogen, South Korea). The resulting sequences were checked for chimeric structures using the Bellerophon program (Huber et al., 2004). Obtained sequences were assigned to species level by using the Identify tool of the EZ Taxon server (Yoon et al., 2017) which also provides global sequence similarities. Clustal X program (Thompson et al., 1997) was used for sequence alignment; maximum likelihood phylogenies were constructed with the Jukes–Cantor distance model and the Kimura two-parameter correction. Phylogenetic trees were built with partial 16S rRNA gene sequences (1350 nt) of the strains, and their close relatives, as selected from the 16S-based ID app within the EZ Taxon server. Resulting dendrograms were tested with a Bootstrap analysis of 1,000 replicates, as implemented in the MEGA6 software package (Tamura et al., 2013).

### Characterization Based on PGPR Traits

For these assays, Standardized Bacterial Inocula (SBI) were obtained by culturing all strains in TY medium (Tryptone 5 g L^–1^, yeast extract 3 g L^–1^, CaCl_2_ 1 g L^–1^), the cultures were incubated at 30°C, with agitation (220 rpm) until log phase was reached. Cultures were centrifuged at 10,000 rpm for 10 min; the pellets were resuspended and washed three times with 0.85% sterile saline solution. Final pellets were resuspended in 5 ml sterile saline and the OD of 1/10 diluted aliquots was measured at 540nm. The suspensions were diluted to reach ˜1.6 × 10^7^ bacteria mL^–1^ according to Lacy and Lukesic (2006). All assays were performed in triplicate and the respective results were compared by ANOVA and a Tukey mean test (*P* ≤ 0.05), with the JMP14 statistical software (JMP, Version 14. SAS Institute Inc.).

#### Production of Indol Acetic Acid (IAA)

Production of IAA (and other indoles) was done in flasks containing 125 mL of Burk medium (Glucose 10 g L^–1^, KH_2_PO_4_ 0.41 g L^–1^, K_2_HPO_4_ 0.52 g L^–1^, NaCl 0.05 g L^–1^, CaCl_2_ 0.2 g L^–1^, MgSO_4_ 7H_2_O 0.1 g L^–1^, FeSO_4_ 0.005 g L^–1^, pH 6.8), amended with 5 mL of tryptophan (11.4 mg mL^–1^). Flasks were inoculated with 1 mL of SBI. Incubation was done under the same conditions, during 144 h, in dark. Quantification of indoles (among them IAA) was done by centrifugation (14,000 rpm for 5 min) of aliquots of each culture. The supernatant was measured and transferred to glass tubes containing Salkowski reagent (H_2_SO_4_ 600 mL, FeCl_3_ 4.5 g, H_2_O enough for 1 L), maintaining a 1:1 ratio between the sample and the reagent. The reactions were incubated in the dark at room temperature for 30 min, then their absorbance at 530 nm was determined (Rives et al., 2009).

#### Phosphate Solubilization

Solubilization of phosphate was done according to Nautiyal (1999). Flasks containing 29 mL of NBRIP liquid medium were inoculated with 1 mL of the respective SBI. Then, the quantification of soluble phosphate following the phospho-molibdate technique was performed. Briefly, 12.5 mL of each culture was centrifuged for 10 min at 14,000 rpm, the supernatants were filtered with Whatman No. 4 filter paper and mixed with 2 mL of the molybdate/ascorbic acid reagent [H_2_SO_4_ 5N, K(SbO) C_4_H_4_O_6_⋅1/2H_2_O 2.74 g L^–1^, (NH_4_)_6_Mo_7_O_24_⋅4H_2_O 40 g L^–1^, 0.1M ascorbic acid]. The reactions were incubated at room temperature for 50 min in the dark and subsequently, their absorbances at 880 nm were determined. The concentrations of soluble phosphate present in the samples under study was calculated according to Murphy and Riley (1962).

#### Siderophores Production

Qualitative tests for the production of siderophores directed against 10 different metals were carried out in LB plates without yeast extract (Peptone 10 g L^–1^, NaCl 10 g L^–1^, agar 15 g L^–1^), supplemented with 1.2 g L^–1^ Chrome Azurol S (CAS), 1.82 g L^–1^ CTAB and 3 mL of the ion to be tested (56 mg L^–1^ in 10 mM HCl). The tested metal ions were: FeCl_2_ (6H_2_O), NiSO_4_⋅6H_2_O, CuCl_2_⋅2H_2_O, K_2_Cr_2_O_7_, ZnSO_4_⋅7H_2_O, NaMoO_4_, NaVO_3_, CdCl_2_, CoCl_2_, and Pb(NO_3_)_2_. Plates were inoculated in triplicate with 30 μL of each SBI, and incubated at 28°C for 72 h. The formation of chelation halos was recorded at 24, 48, and 72 h (Soltani et al., 2012).

#### Biocontrol of Fungal Growth

The ability of bacteria to inhibit the growth of the phytopathogenic fungus *Fusarium solani* was evaluated by the dual culture technique. Petri dishes with PDA medium were inoculated with 20 μL of a spore suspension (2 × 10^7^ spores mL^–1^), in the center of each Petri dish, and 3 cm away, 10 μL of the corresponding SBI were streaked. Petri dishes were incubated from 7 to 15 days at room temperature and the percentage of inhibition exerted by bacteria over the fungi was calculated (Wahyudi et al., 2011).

### Soil Analyses

Soil samples collected at the same sampling points as the rhizospheric samples were used to determine their physico-chemical characteristics. Analyses were carried out at the Laboratory of Edaphology (UAM-Xochimilco), according to the standard procedures described in the national normative (NOM-021-RECNAT, 2000) and Soil Survey (1996).

### Accession Numbers

Massive sequencing data of the cacti rhizosphere metagenome were deposited in Biosample Database under accession numbers: SAMN13482920 to SAMN13482931. 16S rRNA gene sequences of bacteria isolated from *Echinocactus platyacanthus* rhizosphere were deposited in GeneBank under accession numbers: MN062927 to MN062933, MN062936 to MN062939, MN062943, MN062944, MN062946 to MN062948, MN062950 to MN062963, MN443613 to MN443616.

## Results

### Massive Sequencing Data and Parameters of Rhizospheric Bacterial Communities

The output of the Illumina MiSeq system produced 524,604 raw sequences from the 12 rhizospheric samples, their values were ranging from 64,114 to 17,618 raw reads per sample (Supplementary Table S1). After quality evaluation (filtering, trimming, merging and chimera exclusion), the total number was reduced to 213,110 non-chimeric reads. This number of reads was further reduced to 198,652 after the processes of ASV assignation and filtering. Finally, the 12 samples were ranging from 5,663 to 24,528 classified reads per sample (Supplementary Table S1).

Calculated values of α-diversity and species (ASV) richness parameters are given in Table 2. In general, samples in EpC treatment provided the higher values, with EpW values being slightly smaller. However, both groups of samples were quite close, and they did not present significant statistical differences, as compared by one-way ANOVA and Tukey HSD *post hoc* test (*P* ≤ 0.05). In contrast, values for NpW samples were consistently smaller and statistically different from those of the former two treatments (Table 2). Community parameters for NpC treatment showed a different trend, since values related to species richness (observed number of ASVs and the non-parametric estimator Chao1) did not show statistical differences with the other three treatments. However, regarding α-diversity indices (H’ and D), NpC samples were different from NpW (*P* = 0.0126, and 0.0004, respectively), but they were statistically equal to EpC and EpW. Values for Pielou’s evenness index (J) pointed to similarities in rhizospheric bacterial community structure between the two cultivated conditions, EpC and NpC (*P* = 0.979), while values for both wild populations were also similar (*P* = 0.291). Finally, Good’s coverage estimator is equal and quite high (99.99%) for all samples (Table 2), showing that random normalization of the samples to 5213 quality sequences (Supplementary Table S1), almost fully captured their respective total number of ASVs.

**TABLE 2:** Community parameters obtained from Illumina sequencing data of the V6-V8 region of 16S rRNA genes, from rhizospheric samples associated with *E. platyacanthus* (Ep) and *N. polylopha* (Np), cultivated (C) and wild (W). Presented parameters and their standard deviation values are treatment estimators of species richness (Observed ASV, Chao 1), Shannon’s (H’) and Simpson’s (D) diversity indices, and Pielou’s (J) evenness index. Calculations were done after rarefying the respective classified reads (see Supplementary Table S1), to 5,213 sequence reads, corresponding to the smallest number of classified reads in sample NpW-2.

Sample group	Number of observed ASV	Chao1	Shannon H’	Simpson D	Evenness J	Good’s coverage (%)
EpC	758 ± 51^a^	781 ± 61^a^	6.31 ± 0.08^a^	0.9975 ± 0.0003^a^	0.9471 ± 0.0025^a^	99.99
EpW	649 ± 92^a^	666 ± 91^a^	6.08 ± 0.15^a^	0.9963 ± 0.0006^a^	0.9358± 0.0024^ab^	99.99
NpC	528 ± 132^ab^	537 ± 140^ab^	5.95 ± 0.21^a^	0.9963 ± 0.0005^a^	0.9504 ± 0.0056^a^	99.99
NpW	310 ± 110^b^	311 ± 110^b^	5.23 ± 0.32^b^	0.9900 ± 0.0020^b^	0.9193 ± 0.0200^b^	99.99

*Values followed by different letter are statistically significant as tested by one-way ANOVA and Tukey HSD analysis (P-value < 0.05).*

### Composition and Structure of Rhizospheric Bacterial Communities at Different Taxonomic Levels

**FIGURE 1:**
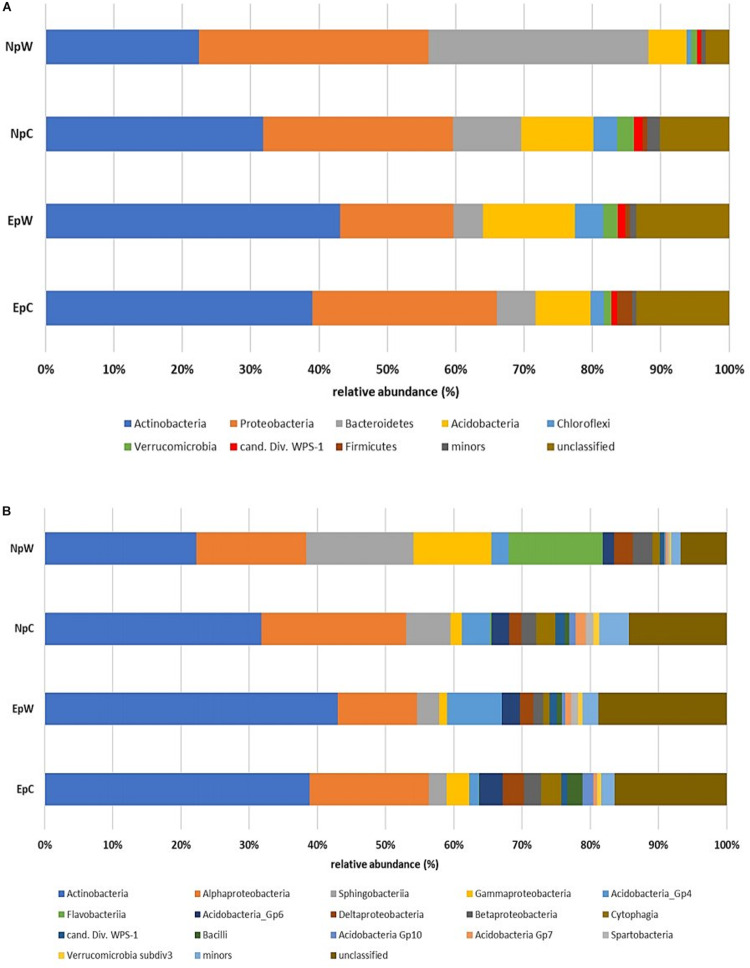
Bacterial community structure in rhizospheric samples of wild and cultivated *E. platyacanthus* and *N. polylopha*. **(A)** Average relative abundances at phylum level, **(B)** Average relative abundances at class level. Only taxa with average relative abundances >0.05% were plotted.

Taxonomic classification of sequences and further analysis at phylum level demonstrated some differences in the structure of the rhizospheric bacterial communities associated with *E. platyacantus* and *N. polylopha.* Regarding composition, all samples accounted for 14 phyla in total, and the 8 major phyla (mean relative abundance >0.5%) were common to all treatments (Figure 1A). The most abundant phylum was *Actinobacteria* ranging from 22% (in NpC) to 43% (in EpW), followed by *Proteobacteria* (16–33%). These two phyla were dominant in all four sample groups and combined they roughly represent 60% of all sequences. The main differences in community structure were detected in NpW samples, where the relative abundance of *Bacteroidetes* (32.1%) was significantly higher than in the other sample groups.

At class level, there were 34 identified classes in all the rhizospheric samples, applying the same criteria as above (mean relative abundance >0.5%), 17 classes were considered as major components (Figure 1B). In this case, not all samples shared these 17 classes. For example, *Flavobacteriia* that was not detected in EpW or with a very low abundance in EpC (0.06%), reached 13.8% relative abundance in samples NpW, thus making *Flavobacteriia* a dominant class (relative abundance >10%) for NpW treatment and significantly higher (*P* = 0.0037) than the other treatments, as assessed by ANOVA and Games-Howell’s test. Interestingly, also the classes *Gammaproteobacteria* and *Sphingobacteriia* showed a similar behavior within this treatment, being dominant classes (11.4 and 15.7% relative abundance, respectively), although only the former was significantly higher (*P* = 0.0006) than the rest of the treatments. Common dominant classes were *Actinobacteria* and *Alphaproteobacteria* that together with *Sphingobacteriia* accounted for 54–59% of all qualified sequences in the four treatments. The relative abundances at phylum and class levels of the 12 rhizospheric samples are depicted in Supplementary Figure S1.

**FIGURE 2:**
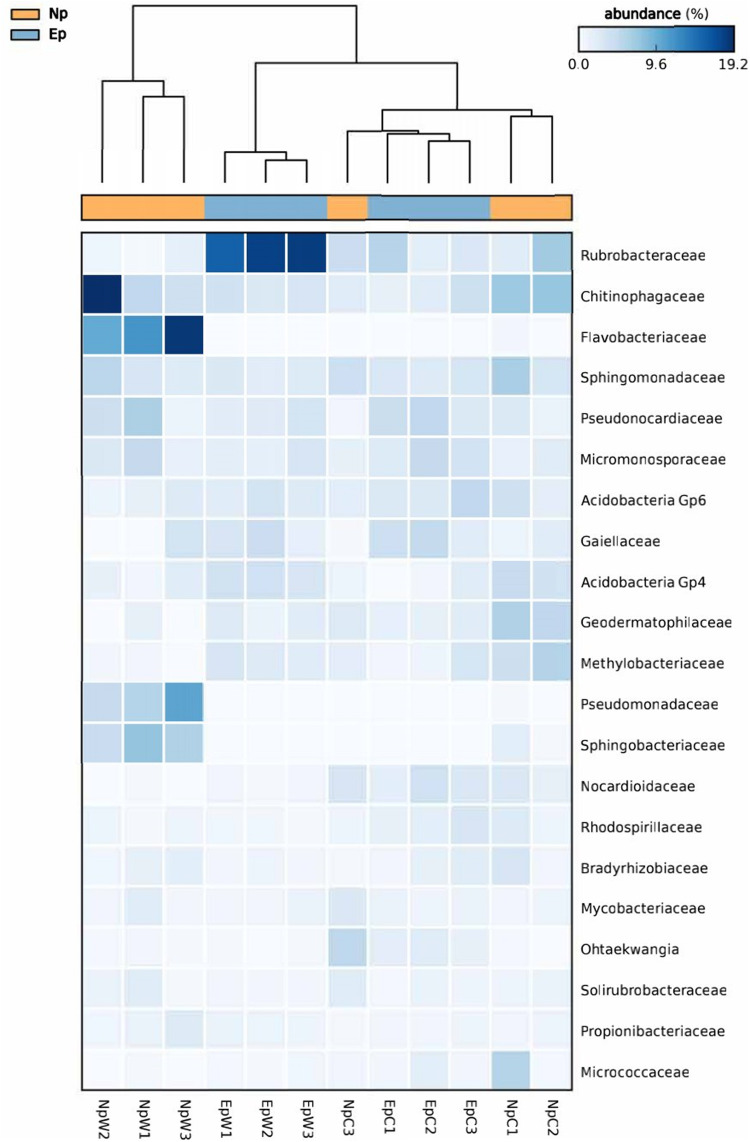
Heatmap based on relative abundances of the top 21 families across all rhizospheric samples; Ep- *E. platyacanthus*, Np- *N. polylopha*, W- wild plants, C- cultivated plants. Abundance color code is depicted in the upper right. Dendrogram in top of the heatmap shows average neighbor clustering of the samples according to their abundance patterns.

Analysis at family level was approached by a heatmap based on the abundances of the top 21 families across all samples (average relative abundances >1%), and further average neighbor (UPGMA) clustering of the samples (Figure 2). At this taxonomical level, the number of unclassified quality reads were ranging from 20.3 to 45.2%. In each group of samples, the respective values of unclassified reads were considered for calculating relative abundances, but family-unclassified reads were not plotted. Although the relative abundances differed greatly among the 12 rhizospheric samples, it was possible to find some abundance patterns between treatments. The high abundance of sequences from *Flavobacteriaceae, Pseudomonadaceae*, and *Sphingobacteriaceae* was a signature in the wild *N. polylopha* (NpW) group of samples. Similarly, the high abundance values of *Rubrobacteraceae* (15.9–18.4%) were a characteristic feature of wild *E. platyacanthus* (EpW) samples. High relative abundances of some families were found in only two out of the three samples per treatment, as in the case of *Chitinophagaceae* and *Geodermatophillaceae* that were scored in two samples of NpC; or *Pseudonocardiaceae* and *Gaiellaceae* in cultivated *E. platyacanthus* (EpC). Although these signatures are graphically noted in the heatmap, the statistical approach used didn’t provide significant differences among groups. The dendrogram on Figure 2, pointed to the EpW samples as the most coherent cluster at family level, according to the distribution of relative abundances. The other wild samples (NpW) also clustered together, with their own pattern distribution that was somehow different from the other treatments. Both sets of cultivated samples (EpC and NpC) formed a homogeneous group.

At the genus level, only 57% of qualified sequences were assigned to 205 genera, however, the average relative abundance of the vast majority (190 genera) was below 1%. The distribution of the top 31 genera, with average abundances >0.5% is depicted in Supplementary Table S2. Among these genera, five of them showed a significant difference; *Rubrobacter* was dominant in EpW, while *Flavobacterium, Pseudomonas*, and *Pedobacter* were major components in samples NpW, and *Streptomyces* was more abundant in EpC treatment.

**FIGURE 3:**
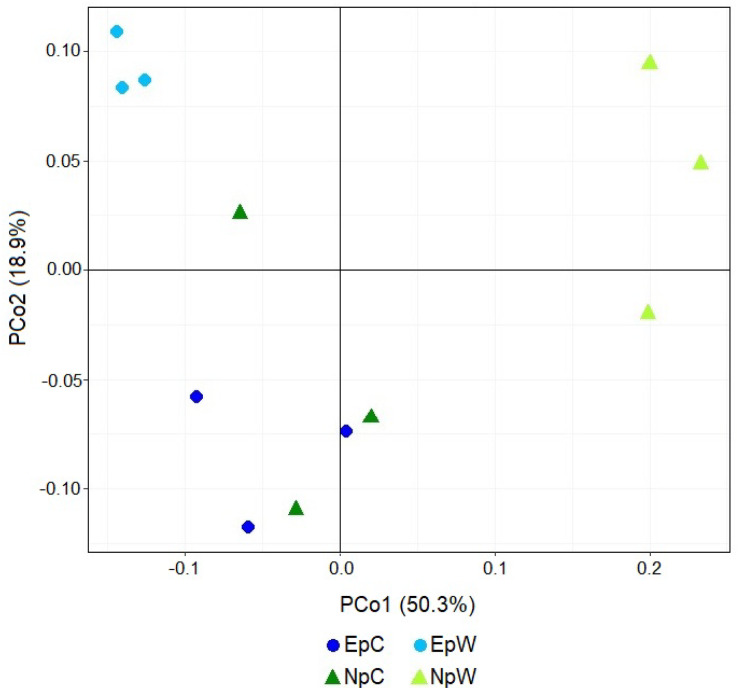
Principal coordinate analysis based on Weighted UniFrac distances of the bacterial genera present in rhizospheric samples: *E. platyacanthus* wild (EpW) and cultivated (EpC); *N. polylopha*. wild (NpW) and cultivated (NpC).

ASV composition analysis of the bacterial communities was assessed by PCoA (Figure 3). Under this approach, wild samples from both plant species formed two clear clusters, with the 3 EpW samples making a very compact group, while the 3 NpW samples were less close but clearly separated from all other samples. The 6 cultivated samples (EpC and NpC) were intermingled forming a less coherent group, which is reflecting the varied origins of the cultivated samples. These results based on Weighted UniFrac distances from the relative abundance matrix were tested by PERMANOVA. The results indicated that the wild group of samples (EpW and NpW) were significantly different (*P* = 0.017) than the cultivated ones. This was further assessed with BETADISPER producing a non-significant value, and thus indicating that differences in wild vs. cultivated samples are not due to the beta-dispersion of the replicates of each sample.

**FIGURE 4:**
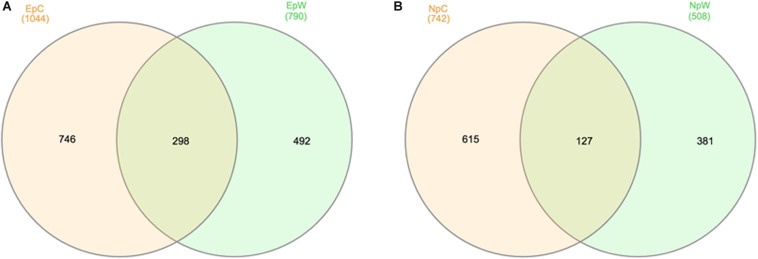
Venn diagrams showing the overlap and distribution of common and exclusive ASVs calculated for each cactus species. **(A)** *E. platyacanthus* wild (EpW) and cultivated (EpC); **(B)** *N. polylopha* wild (NpW) and cultivated (NpC). ASVs scoring in only one out of the six samples per cactus species were not considered.

The initial 213,110 non-chimeric reads (see Supplementary Table S1) were first assigned into 6,804 ASVs, but after removing low abundance, plastid and unclassified (domain level) sequences, there were 2,227 ASVs left from all 12 rhizospheric samples. Venn diagram analysis pointed at the shared and exclusive ASVs for each plant species and growing condition (Figure 4). Ep samples (Figure 4A) showed a higher richness with 1,536 ASVs, while Np samples (Figure 4B) only had 1,123. Regarding the growing conditions, the cultivated samples (EpC and NpC) were also richer than their wild counterparts accounting for 746 and 615 ASVs, respectively. From the 1044 ASV present in EpC, 746 (71%) were present only in these samples, accounting for 62.3% of average relative abundance, while the 492 out of 790 ASV (62%) belonging exclusively to EpW retained 44.5%, pointing to a more uneven ASV distribution of EpW samples, as they have many ASV with very low relative abundance. On the side of Np samples this feature was somehow the opposite, since the 615 EpC exclusive ASV (83% of 742 ASV) represented only 64.3% of the average relative abundance, while for NpW 75% of the exclusive ASV (382) retained 71.6% average relative abundance. Furthermore, from shared ASV, in Ep 15 of them were present in all six samples, representing 7.0 and 8.2% of average relative abundance for EpC and EpW, respectively. For Np samples, only 4 out of 127 shared ASV were scored in all six samples, but in NpC they conserved 3.4% average relative abundance, the respective value for NpW was 3.9%.

### Microbiological Analyses of *Echinocactus platyacanthus* Samples

The results obtained from the CFU counts for EpW samples were 4.27 × 10^10^ ± 5.2 × 10^9^ and for EpC samples were 1.06 × 10^6^ ± 5.12 × 10^5^ CFU g^–1^ of rhizospheric soil. Although there was a difference of almost four orders of magnitude in the CFU values, Tukey test (*p* ≤ 0.05) showed no statistically significant differences between the two samples.

**FIGURE 5:**
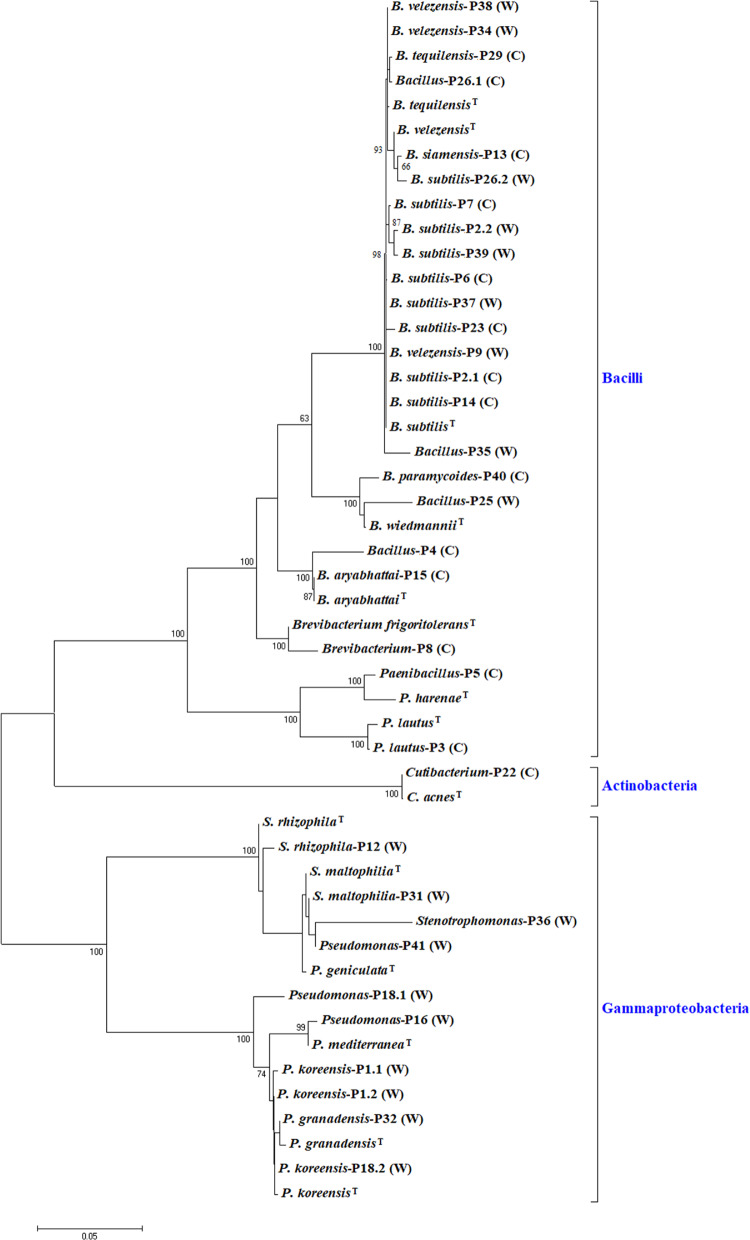
Phylogenetic tree based on nearly full length 16S rRNA gene sequences (1350 nt), of bacteria isolated from *Echinocactus platyacanthus* rhizosphere. The dendrogram was built with the Maximum Likelihood method and the Jukes-Cantor substitution model. The topology was tested by Bootstrap analysis using 1000 randomizations, only bootstrap values greater than 60% are shown.

In total, 194 bacterial strains were isolated from the wild and cultivated Ep samples. They were further grouped by ARDRA into 41 ribotypes (P1–P41), based on the assumption that the strains within the same ribotype should be the same or related isolates (Supplementary Table S3). Strains identification and phylogenetic analysis were based on nearly full-length 16S rRNA gene sequences. For this purpose, one isolate from each ARDRA pattern and two strains for those patterns containing 8 or more isolates (P1, P2, P18, and P26) were selected. The taxonomic identification of the bacteria representing each ribotype was performed with the 16S-based ID app at the EZ Biocloud webserver (Yoon et al., 2017) whose curated database contains only sequences of type strains and known isolates. It was possible to identify 35 out of the 44 strains under study. At the genus level, 11 strains were identified based on sequence similarities ranging between 95 and 98.6% (P4, P5, P8, P16, P22, P25, P26, P28, P35, P36, and P41) (Bou et al., 2011). According to Kim et al. (2014) strains with a similarity percentage greater than 98.7% were identified at the species level (see Supplementary Table S3). Similarity scores for strains P10, P11, P17, P19, P20, P21, P24, P27, P30, and P33 were <95%. Thus, the 18 isolates included in these ribotypes were excluded from further analysis. The dendrogram in Figure 5 shows the phylogenetic relationships of the identified strains and their closest type strains. Almost all the isolates belonged to the classes *Bacilli* and *Gammaproteobacteria*, and they were distributed in 3 and 2 genera, respectively.

Comparison of the V6–V8 region of the 16S rRNA gene of the isolates to the corresponding ASV sequences, allowed the correct assignment of 13 ribotype sequences to 5 specific ASVs (Supplementary Table S4). The sequences of 8 isolates (ribotypes P6, P9, P13, P14, P26.2, P34, P37, P38) identified as different *Bacillus* species completely matched to ASV0805 (*Bacillus*). In spite of the high abundance (12.5%) of this sequence fragment among the collection of isolates (present in 22 out of 176 isolates), this ASV had a relative abundance of 0.027% and was only found in EpC samples. The ribotype P32 comprising one strain (*Pseudomonas granadiensis* P32) was assigned to ASV0008 (*Pseudomonas*), which had a high relative abundance of 0.42%, placing this ASV among the top 10 more abundant. The other 4 correctly assigned ribotype sequences matched to ASVs with very low abundance (<0.005%), that were excluded after filtering. The remaining 28 tested sequences did not match exactly with any of the existing ASVs.

In a search for isolates with abilities to promote plant growth, all identified strains were assayed for the following PGPR traits; production of indoles, solubilization of phosphate, production of siderophores against 10 different metals, and growth inhibition over the phytopathogenic fungi *Fusarium solani*. The results obtained are shown in Table 3, the values of the 3 quantitative assays (production of indoles, phosphate solubilization, and fungal growth inhibition) were subject to ANOVA, followed by *post hoc* Tukey tests. All strains were producers of indoles (including IAA) and phosphate solubilizers, ranging from 12.98 to 92.52 μg mL^–1^, and 3.15 to 75.39 μg mL^–1^, respectively. From the 35 tested strains, 22 of them exerted growth inhibition against *Fusarium solani*, and 14 of these strains can be considered good inhibitors (>45% growth inhibition).

**TABLE 3:** PGPR traits of selected bacterial strains representing the 41 ribotype-groups of isolates obtained from *Echinocacthus platyacanthus* rhizosphere.

Origin	Strain	Indoles production (μg mL^–1^)	Phosphate solubilization (P-PO_4_ mL^–1^)	Inhibition of *Fusarium* (%)	Production of siderophore vs. different metal ions									
					Fe	Ni	Cu	K	Zn	Mo	V	Cd	Co	Pb
Wild	*Bacillus* (P25)	21.220.37^*rs*^	15.520.49^fgh^	39.172.89^ef^	−	−	−	−	−	−	−	−	−	−
	*Bacillus* (P35)	57.920.62^j^	27.256.69^de^	49.171.44^de^	−	−	−	−	−	−	−	−	−	−
	*B. subtilis* (P2.2)	19.230.58^st^	66.831.76^b^	0	+	−	+	−	+	−	+	+	−	−
	*B. subtilis* (P37)	66.660.16^g^	16.473.22^fgh^	0	−	−	−	−	−	−	−	−	−	−
	*B. subtilis* (P39)	17.780.26^*t*^	15.971.49^fgh^	0	−	−	−	−	−	−	−	−	−	−
	*B. subtilis* (P26.2)	20.140.28^*rs*^	2.731.56^j^	0	+	+	+	+	+	+	+	+	+	+
	*B. velezensis* (P34)	64.27.041^h^	15.741.83^fgh^	41.673.82^ef^	−	−	−	−	−	−	−	−	−	−
	*B. velezensis* (P38)	81.220.69^c^	18.372.07^fg^	0	−	−	−	−	−	−	−	−	−	−
	*B. velezensis* (P9)	29.280.73^p^	18.241.08^fg^	78.332.89^a^	−	−	−	−	−	−	−	−	−	−
	*Pseudomonas* (P16)	68.090.26^fg^	17.702.03^fg^	0	−	−	−	−	−	−	−	−	−	−
	*Pseudomonas* (P18.1)	66.880.65^g^	11.400.26^ghi^	34.171.44^f^	−	−	−	−	−	−	−	−	−	−
	*Pseudomonas* (P41)	36.610.30^n^	65.081.54^b^	49.171.44^de^	+	+	+	+	+	+	+	+	+	+
	*P. granadensis* (P32)	76.261.47^d^	20.122.68^ef^	34.171.44^f^	−	−	−	−	−	−	−	−	−	−
	*P. koreensis* (P1.1)	77.620.32^d^	3.610.86^j^	0	−	−	−	−	−	−	−	−	−	−
	*P. koreensis* (P1.2)	50.431.59^*kl*^	68.861.52^ab^	47.502.5^de^	+	+	+	+	+	+	+	+	+	+
	*P. koreensis* (P18.2)	24.520.75^*q*^	71.381.56^ab^	73.331.44^ab^	+	−	+	−	−	−	+	+	−	−
	*Stenotrophomonas* (P36)	37.050.32^n^	17.022.35^fgh^	49.171.44^de^	−	−	−	−	−	−	−	−	−	−
	*S. maltophilia* (P31)	58.440.41^j^	4.960.79^ij^	47.502.5^de^	+	+	+	+	+	+	+	+	+	+
	*S. rhizophila* (P12)	92.520.90^a^	36.124.76^c^	61.675.20^c^	−	−	−	−	−	−	−	−	−	−
Cultivated	*Bacillus* (P26.1)	13.670.43^u^	18.006.49gf	41.880.76^ef^	−	−	−	−	−	−	−	−	−	−
	*Bacillus* (P28)	60.990.74^i^	6.400.13^ij^	54.173.82^cd^	−	−	−	−	−	−	−	−	−	−
	*Bacillus* (P4)	48.830.22^*l*^	3.150.56^j^	54.482.75^cd^	−	−	−	−	−	−	−	−	−	−
	*B. aryabhattai* (P15)	46.170.65^*m*^	20.651.66^ef^	0	−	−	−	−	−	−	−	−	−	−
	*B. paramycoides* (P40)	83.630.66^b^	75.390.63^a^	0	−	−	−	−	−	−	−	−	−	−
	*B. siamensis* (P13)	67.570.68^fg^	9.770.67^hij^	0	−	−	−	−	−	−	−	−	−	−
	*B. subtilis* (P2.1)	33.550.48^o^	28.872.63^cd^	0	−	−	−	−	−	−	−	−	−	−
	*B. subtilis* (P14)	30.330.32^p^	19.355.05^f^	0	−	−	−	−	−	−	−	−	−	−
	*B. subtilis* (P23)	67.980.38^fg^	6.260.83^ij^	45.837.22^de^	−	−	−	−	−	−	−	−	−	−
	*B. subtilis* (P6)	91.320.92^a^	5.521.09^ij^	41.673.82^ef^	−	−	−	−	−	−	−	−	−	−
	*B. subtilis* (P7)	51.080.06^*k*^	7.330.83^ij^	0	−	−	−	−	−	−	−	−	−	−
	*B. tequilensis* (P29)	35.570.08^n^	7.390.92^ij^	64.172.89^bc^	−	−	−	−	−	−	−	−	−	−
	*Brevibacterium* (P8)	72.010.02^e^	5.231.37^ij^	40.832.89^ef^	−	−	−	−	−	−	−	−	−	−
	*Cutibacterium* (P22)	13.420.32^u^	11.650.41^ghi^	47.500^de^	−	−	−	−	−	−	−	−	−	−
	*Paenibacillus* (P5)	21.040.32^*rs*^	3.480.91^j^	54.211.39^cd^	−	−	−	−	−	−	−	−	−	−
	*P. lautus* (P3)	21.990.77^*r*^	15.700.99^fgh^	48.679.42^de^	−	−	−	−	−	−	−	−	−	−

**Different superscript letters denote groups of statistically significant values, according to ANOVA, followed by Tukey tests.**

Interestingly, most of the strains showing a high value in any of the PGPR traits assayed (those with a-d letters, in Table 3), as well as all the siderophore producers, belonged to wild *E. platyacanthus* samples. Among this group with good PGPR activities, *Stenotrophomonas rhizophila* strain P12 and *Pseudomonas koreensis* strains P1.2 and P18.2 were of particular interest as the former has three top values for IAA production, phosphate solubilization and fungal growth inhibition albeit they do not produce any siderophore. Both *P. koreensis* strains showed top values for phosphate solubilization and are good fungi inhibitors. Additionally, they produce siderophores. From all tested isolates, *P. koreensis* strain P1.2 produced the largest chelation halos against all metal ions after 48 h (data not shown).

## Discussion

The rhizosphere microbial communities play important roles in the adaptation of the plants to their different environments (Bulgarelli et al., 2013). These also include plants growing in arid and semi-arid environments, and their associated microbial communities (Fonseca-García et al., 2018). In this study, the diversity and structure of the rhizospheric bacterial communities associated with the cactus plants, *E. platyacanthus* and *N. polylopha* growing in natural populations at the Queretaro semi-desert were assessed, and compared to the rhizospheric communities of the same cacti species growing under cultivated conditions, either in nurseries or in gardens.

According to our initial observation, the major bacterial classes detected in all sample groups were *Actinobacteria, Gammaproteobacteria, Alphaproteobacteria*, and *Sphingobacteria* (>5% average abundance). Some other classes like *Bacilli* and *Cytophagia* were also present in our samples, but they were considered as minor components. Previous studies have already reported several genera and species within those classes in association with other *Cactaceae*. Puente et al. (2004) have described *Bacillus* (*Bacilli*) and *Pseudomonas* (*Gammaproteobacteria*) as rock-weathering bacteria associated with the cacti *Pachycereus pringlei* and *Opuntia cholla*. Furthermore, these authors (Puente et al., 2009) enlarged the list of rock-degrading bacterial genera with the addition of *Staphylococcus* (*Bacilli*), *Klebsiella* and *Acinetobacter* (*Gammaproteobacteria*), that were obtained as root and seed endophytes from *P. pringlei*. Similarly, members of many genera within the class *Actinobacteria* have also been reported in association with *Cactaceae* (Aguirre-Garrido et al., 2012; Andrew et al., 2012; Lee and Seong, 2014; Bustillos-Cristales et al., 2017) or as common plant-associated bacteria. These bacteria are known to play several ecological roles, due to the wide variety of metabolites produced by them (Bulgarelli et al., 2013). In our rhizospheric samples, sequences within the genus *Bacillus* were detected among the top 30 abundant genera (>0.5%) in Ep samples. *Pseudomonas*-related sequences were also found as a major genus in Np samples, particularly in the NpW representing almost 7% abundance. Actinobacteria was the most abundant phylum and class in all our groups of samples. At the genus level, 12 out of the 31 abundant genera belonged to these taxonomic groups, with genera like *Rubrobacter* and *Streptomyces* being main components in the structure of the Ep bacterial communities.

Moreover, analyses at phylum and class levels showed that the bacterial communities in our samples were very similar, sharing most of the major taxa, with slight variations according to the plant species. However, family-heatmap analysis and PCoA at ASV level (Figures 2, 3, respectively) demonstrated that *E. platyacanthus* and *N. polylopha* wild plants have their own characteristic rhizospheric communities. It was also noted that the structure of bacterial communities in the cultivated plants of both species was similar among them, and statistically not different. Furthermore, diversity and ASV richness of rhizospheric bacterial communities in cultivated plants from both cacti species was higher than their wild-grown counterparts. The report of Coleman-Derr et al. (2016) pointed to an opposite effect in case of cultivated *Agave tequilana*, and its putative wild counterparts *A. salmiana* and *A. deserti* that showed higher fungal and bacterial diversity. However, this apparent contradiction may be explained by the wider origin of our cultivated samples.

Plant-associated bacterial communities are probably influenced by several environmental factors such as geographic location, seasonal effects, soil texture, and chemistry, etc. There are as well, host-related factors including plant compartment, phenotype and genotype. Among the climate and soil features of Huajales sampling site (Table 1), the values of precipitation, total porosity, and organic matter content were higher compared to all other samples taken from Toliman and surroundings. Interestingly, these milder environmental conditions at Huajales were not reflected in NpW samples, as they showed the lowest diversity and ASV richness of all sampling groups. Soil physicochemical characteristics of NpC, EpW, and EpC samples were very similar among them. The importance of the habitat as the main force in shaping the plant-associated bacterial communities has been already pointed for other plants such as maize (Peiffer et al., 2013) rice (Edwards et al., 2015) and within the family *Cactaceae*, for *Myrtillocactus geometrizans* and *Opuntia robusta* (Fonseca-García et al., 2016). Bacterial communities in *E. platyacanthus* rhizosphere may also be influenced by host-related factors. In spite of the differences in soil, climate, and the vast distance between both sampling sites (ca 65 km); EpC and EpW shared almost 30% of their ASV. The differences in microbial community structure between wild and cultivated plants have been reported for different species of *Agave* (Coleman-Derr et al., 2016; Fonseca-García et al., 2018) suggesting that cultivation of *A. tequilana* negatively influences its microbial diversity. A recent report (Flores-Nuñez et al., 2020) approached by shotgun metagenome analysis, pointed again in the same direction; the rhizospheric bacterial community of *A. tequilana* was less diverse than that of wild *A. salmiana*, and other wild cacti. Although their cultivated and native non-rhizospheric soil samples showed similar diversities. Our study is the first report stressing the differences in the rhizospheric bacterial communities of wild and cultivated cacti from the same species. This research also points to a different direction than previous works on non-cactus plants, in the sense that we have found higher diversities in the bacterial communities of cultivated plants.

Under the culture-based approach, a collection of 194 rhizospheric isolates was grouped in 41 ARDRA ribotypes, and representatives of these groups were identified by 16S rRNA gene sequencing. Comparison of the respective sequence fragments of the isolates to the corresponding ASVs data, provided by Illumina sequencing, pointed that partial sequences assigned into one ASV may belong to individuals from different species according to nearly full-length 16S rRNA gene sequence. Furthermore, in general there is a lack of correlation between the abundance of isolates and the relative abundance of the corresponding sequences, as detected by NGS methods. This fact has already been reported in banana sprouts by Thomas who found that several predominant genera according to the metagenomic profile were not recovered in culture, while some of the abundantly cultivated genera were recorded as minor constituents by massive sequencing.

Adverse environmental conditions in ASZ include low water availability, eroded-saline soils, and high Sun-radiation levels and temperature (González-Medrano, 2012; Granados-Sánchez et al., 2013; Landa et al., 2014) imposing a challenge for plant establishment. Furthermore, upon establishment, xerophytic plants in ASZ may also require extra bacterial metabolic activities helping to improve their growth. Table 4 depicts information on 10 bacterial genera that were highly detected in our rhizospheric samples (>0.5% average relative abundance). These genera have already been reported with some adaptive activity to extreme conditions or as a PGPR, and the data gathered were especially focused on plants from ASZ. The presence and abundance of these and the above-mentioned bacterial genera may suggest that they can also play important roles in the ecology and survival of *E. platyacanthus* and *N. polylopha*.

**TABLE 4:** Reported metabolic or PGPR activities of rhizospheric bacteria associated with plants growing in ASZ, these functions may play a role in the adaptation to arid and semi-arid environments.

Genus	Metabolic and/or PGPR activities	Associated with	References	Found in samples
*Arthrobacter*	Halotolerant; hydrocarbon degradation; N fixation; cytokinin production; P solubilization	*Mammilaria carnea, Opuntia pilifera*	Aguirre-Garrido et al., 2012; Hamedi et al., 2015; Baliyarsingh et al., 2017; Gangwar et al., 2017; Pradhan et al., 2017	Ep/Np
*Gaiella*	Halotolerant	*Boswellia sacra*	Yang et al., 2016; Al-Harrasi et al., 2019	Ep/Np
*Geodermatophilus*	Halotolerant; nitrate reduction; organic acid release; lytic enzyme activity (acid and alkaline phosphatase, lipase, and protease)	*Cereus jamacaru*	Zhang et al., 2011; Kavamura et al., 2013; Montero-Calasanz et al., 2013a, b	Ep/Np
*Microvirga***^§^**	N fixation; nodule formation; nitrate reduction; organic acid release; catalase, urease and tryptophan deaminase activities	*Listia angolensis, Lupinus texensis*	Ardley et al., 2012	Ep/Np
*Ohtaekwangia*	Acid and alkaline phosphatase activities; hydrocarbon degradation	*Boswellia sacra*	Yoon et al., 2011; Hou et al., 2015; Al-Harrasi et al., 2019	Ep/Np
*Pedobacter***^§^**	Biocontrol of pathogenic bacteria	No data available	Wong et al., 2011	Np
*Pseudomonas*	IAA production; ACC deaminase activity; phytopathogen biocontrol	*Boswellia sacra, Mammilaria carnea, Opuntia pilifera, Prosopis articulata*	Couillerot et al., 2009; Aguirre-Garrido et al., 2012; Galaviz et al., 2018; Al-Harrasi et al., 2019	Np
*Rubrobacter*	Solubilization of minerals; thermophilic; halotolerant; nitrate reduction; organic acid release; oxidase and catalase activities	*Mammilaria carnea*, *Cereus jamacaru, Boswellia sacra, Prosopis articulata*	Torres-Cortés et al., 2012; Kavamura et al., 2013; Kämpfer et al., 2014; Baliyarsingh et al., 2017; Galaviz et al., 2018; Al-Harrasi et al., 2019	Ep/Np
*Solirubrobacter*	Enzyme activity (acid and alkaline phosphatase, oxidase, catalase, and protease)	*Prosopis articulate*	Kim et al., 2007; Galaviz et al., 2018	Ep/Np
*Sphingomonas*	Halotolerant; N fixation; denitrification; IAA and gibberellic acid production	*Boswellia sacra, Prosopis articulate*	Maheshwari et al., 2015; Yang et al., 2016; Gangwar et al., 2017; Galaviz et al., 2018; Al-Harrasi et al., 2019	Ep/Np

**Sequences of these genera were abundant in one or both of our rhizospheric samples (last column).****^§^****There are no available reports of these bacterial genera in association with plants growing in ASZ.**

Currently, about 30% of the Mexican cactus species are exposed to a risk or are under a threatening category to their survival (NOM – 059, IUCN, 2019) thus protection measures are urgently needed. At present, the structure of wild populations of *E. platyacanthus* at several spots of the Chihuahuan desert is suffering from lack of plantlets and young cacti (Jiménez-Sierra and Matías-Palafox, 2015). Thus, the nurseries propagating this and other cactus species are the main source of young specimens. The knowledge about the rhizospheric bacterial communities in these cacti, as well as the search for PGPR associated with them, may help to improve cacti production in the nurseries, and the fitness of plants when re-introduction measures are taken.

## Data Availability Statement

The datasets generated for this study can be found in the Biosample Database, accession numbers: SAMN13482920 to SAMN13482931, GeneBank under accession numbers: MN062927 to MN062933, MN062936 to MN062939, MN062943, MN062944, MN062946 to MN062948, MN062950 to MN062963, MN443613 to MN443616.

## Author Contributions

MT-H contributed with the sample collection and experiments, also writing and correction of manuscript. LS-V contributed with the experiments, writing, and formatting of manuscript. JA-G carried out data analysis. AF-G carried out the bioinformatics and statistical analyses. FM-A contributed with discussion and correction of manuscript. DM-L carried out the sample collection and lab processing. HR-S conceived and designed the study, carried out the data analysis, writing, discussion, and correction of the manuscript. All authors contributed to the article and approved the submitted version.

## Conflict of Interest

The authors declare that the research was conducted in the absence of any commercial or financial relationships that could be construed as a potential conflict of interest.

## Footnotes

1 https://benjjneb.github.io/dada2/tutorial.html

2 https://mafft.cbrc.jp/alignment/server/

3 http://www.interactivenn.net/

## Supplementary Material

FIGURE S1 Community structure of samples.

TABLE S1 Massive sequencing data.

TABLE S2 Genus Profile Top31.

TABLE S3 Strain identification.

TABLE S4 Isolates vs. ASV.
