# Supplementary figures and images for: Composition, Structure, and PGPR Traits of the Rhizospheric Bacterial Communities Associated With Wild and Cultivated Echinocactus platyacanthus and Neobuxbaumia polylopha
Source: Front Microbiol. 2020 Jun 26;11:1424. doi: 10.3389/fmicb.2020.01424 (PMC7333311; doi:10.3389/fmicb.2020.01424)

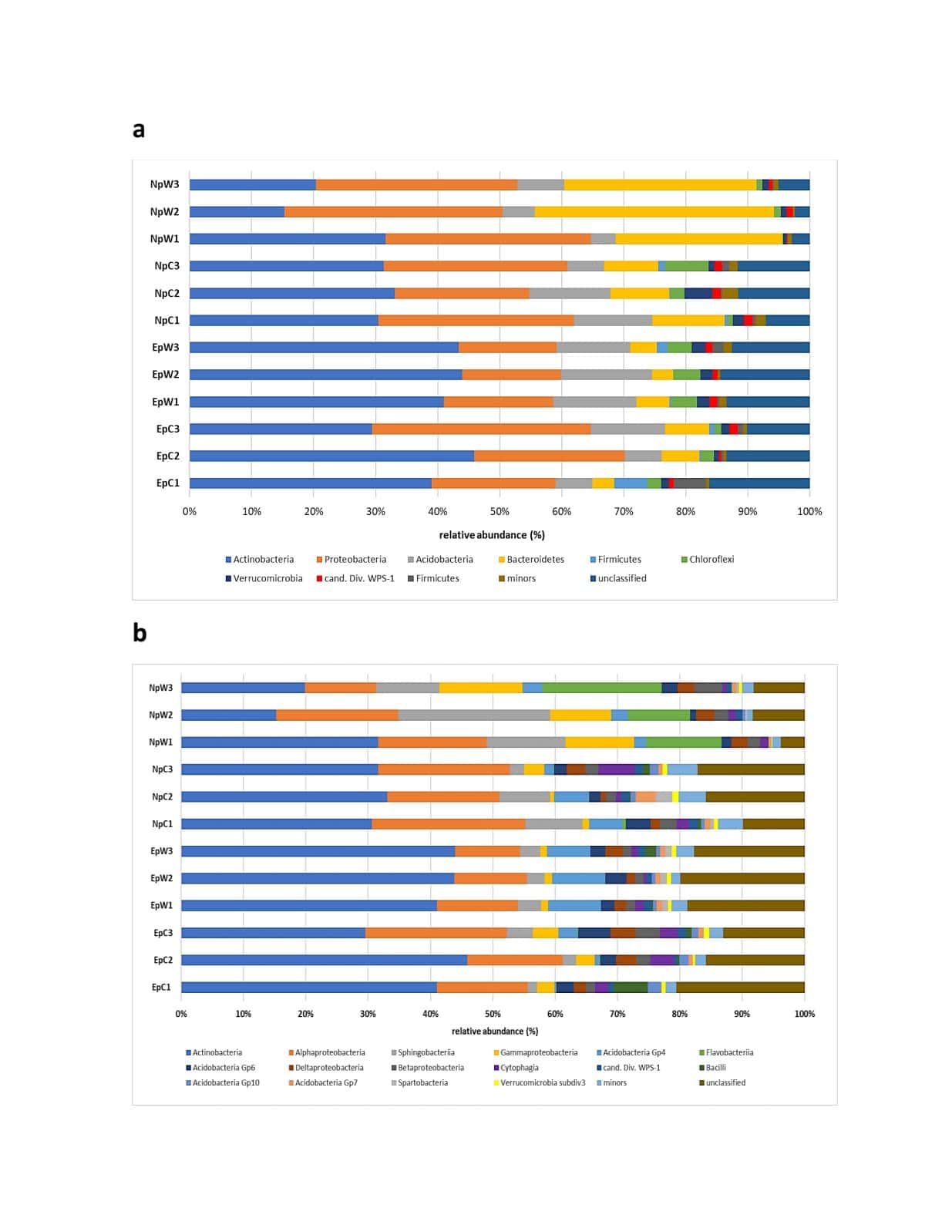

Supplement: FIGURE S1 — Community structure of samples. [file Image_1.TIF]
