# Supplementary material for: Composition, Structure, and PGPR Traits of the Rhizospheric Bacterial Communities Associated With Wild and Cultivated Echinocactus platyacanthus and Neobuxbaumia polylopha
Source: Front Microbiol. 2020 Jun 26;11:1424. doi: 10.3389/fmicb.2020.01424 (PMC7333311; doi:10.3389/fmicb.2020.01424)
Supplement: TABLE S1 — Massive sequencing data. [file Table_1.DOCX]

**Table S1**. Characteristics of the Illumina miSeq sequencing data of the 12 rhizospheric samples. Values in each column represent the number of reads after each successive step of quality-filtering. The last column (Classified) correspond to the number of reads left after assigning ASV, removing low abundance (<0.005%), and plastid sequences.

| **Samples** | **Raw reads** | **Filtered reads** | **Denoised R1** | **Denoised R2** | **Merged** | **No chimeric** | **Classified** |
| --- | --- | --- | --- | --- | --- | --- | --- |
| EpC-1 | 42276 | 36875 | 34256 | 35448 | 27178 | 24057 | 21970 |
| EpC-2 | 43930 | 38119 | 35372 | 36468 | 27975 | 24528 | 22843 |
| EpC-3 | 39891 | 35321 | 32889 | 33854 | 26930 | 23913 | 21837 |
| EpW-1 | 59838 | 47962 | 42261 | 45166 | 26127 | 21993 | 20624 |
| EpW-2 | 64114 | 43098 | 37726 | 40566 | 22819 | 19234 | 18501 |
| EpW-3 | 57165 | 44371 | 38949 | 41514 | 23907 | 20374 | 19299 |
| NpC-1 | 34293 | 27370 | 24320 | 25847 | 17100 | 14794 | 13796 |
| NpC-2 | 51500 | 41262 | 36468 | 39237 | 24717 | 20868 | 19370 |
| NpC-3 | 35086 | 28038 | 23707 | 25520 | 13357 | 11316 | 10511 |
| NpW-1 | 37864 | 29572 | 25097 | 27377 | 13815 | 11484 | 10652 |
| NpW-2 | 17618 | 14041 | 11312 | 12545 | 6664 | 5663 | 5213 |
| NpW-3 | 41029 | 33321 | 28326 | 30800 | 17409 | 14886 | 14036 |
| TOTAL | 524604 | 419350 | 370683 | 394342 | 247998 | 213110 | 198652 |
