# Supplementary material for: Composition, Structure, and PGPR Traits of the Rhizospheric Bacterial Communities Associated With Wild and Cultivated Echinocactus platyacanthus and Neobuxbaumia polylopha
Source: Front Microbiol. 2020 Jun 26;11:1424. doi: 10.3389/fmicb.2020.01424 (PMC7333311; doi:10.3389/fmicb.2020.01424)
Supplement: TABLE S3 — Strain identification. [file Table_3.DOCX]

| **Table S3.** Identification of rhizospheric isolates obtained from *Echinocactus platyacanthus.* | | | | |
| --- | --- | --- | --- | --- |
| **Pattern (number of isolates)** | **Strain** | **Accession**  **number** | **Similarity**  **(%)** | **Closest type sequence** |
| P1 (42) | *Pseudomonas koreensis* (P1.1) | MN443613 | 99.7 | *Pseudomonas koreensis* (AF468452) |
|  | *Pseudomonas koreensis* (P1.2) | MN062960 | 99.57 | *Pseudomonas koreensis* (AF468452) |
| P2 (47) | *Bacillus subtilis* ssp. *inaquosorum* (P2.1) | MN443614 | 100 | *Bacillus subtilis* ssp. *inaquosorum* (AMXN01000021) |
|  | *Bacillus subtilis* ssp. *inaquosorum* (P2.2) | MN062961 | 99.23 | *Bacillus subtilis* ssp. *inaquosorum* (AMXN01000021) |
| P3 (4) | *Paenibacillus lautus* (P3) | MN062927 | 99.42 | *Paenibacillus lautus* (D78473) |
| P4 (4) | *Bacillus* (P4) | MN062928 | 95.23 | *Bacillus aryabhattai* (EF114313) |
| P5 (4) | *Paenibacillus* (P5) | MN062929 | 96.22 | *Paenibacillus harenae* (AY839867) |
| P6 (1) | *Bacillus subtilis* ssp. *inaquosorum* (P6) | MN062930 | 100 | *Bacillus subtilis* ssp. *inaquosorum* (AMXN01000021) |
| P7 (5) | *Bacillus subtilis* ssp. *inaquosorum* (P7) | MN062931 | 99.65 | *Bacillus subtilis* ssp. *inaquosorum* (AMXN01000021) |
| P8 (2) | *Brevibacterium* (P8) | MN062932 | 95.77 | *Brevibacterium frigoritolerans* (AM747813) |
| P9 (4) | *Bacillus velezensis* (P9) | MN062933 | 99.14 | *Bacillus velezensis* (AY603658) |
| P12 (4) | *Stenotrophomonas rhizophila* (P12) | MN443615 | 100 | *Stenotrophomonas rhizophila* (CP007597) |
| P13 (2) | *Bacillus siamensis* (P13) | MN062936 | 99.55 | *Bacillus siamensis* (AJVF01000043) |
| P14 (4) | *Bacillus subtilis* ssp. *inaquosorum* (P14) | MN062937 | 99.86 | *Bacillus subtilis* ssp. *inaquosorum* (AMXN01000021) |
| P15 (1) | *Bacillus aryabhattai* (P15) | MN062938 | 99.65 | *Bacillus aryabhattai* (EF114313) |
| P16 (1) | *Pseudomonas* (P16) | MN062939 | 98.65 | *Pseudomonas mediterranea* (AUPB01000004) |
| P18 (15) | *Pseudomonas* (P18.1) | MN443616 | 99.7 | *Pseudomonas koreensis* (AF468452) |
|  | *Pseudomonas koreensis* (P18.2) | MN062962 | 99.72 | *Pseudomonas koreensis* (AF468452) |
| P22 (3) | *Cutibacterium* (P22) | MN062943 | 97.89 | *Cutibacterium acnes* (AWZZ01000008) |
| P23 (1) | *Bacillus subtilis* ssp. *inaquosorum* (P23) | MN062944 | 99.57 | *Bacillus subtilis* ssp. *inaquosorum* (AMXN01000021) |
| P25 (1) | *Bacillus* (P25) | MN062946 | 97.57 | *Bacillus wiedmannii* (LOBC01000053) |
| P26 (8) | *Bacillus* (P26.1) | MN062947 | 99.56 | *Bacillus velezensis* (AY603658) |
|  | *Bacillus subtilis* ssp. *subtilis* (26.2) | MN062963 | 99.32 | *Bacillus subtilis* ssp. *subtilis* (ABQL01000001) |
| P29 (1) | *Bacillus tequilensis* (P29) | MN062948 | 99.51 | *Bacillus tequilensis* (AYTO01000043) |
| P31 (1) | *Stenotrophomonas maltophilia* (P31) | MN062950 | 99.57 | *Stenotrophomonas maltophilia* (JALV01000036) |
| P32 (1) | *Pseudomonas granadensis* (P32) | MN062951 | 99.08 | *Pseudomonas granadensis* (LT629778) |
| P34 (3) | *Bacillus velezensis* (P34) | MN062952 | 98.72 | *Bacillus velezensis* (AY603658) |
| P35 (1) | *Bacillus* (P35) | MN062953 | 98.13 | *Bacillus subtilis* ssp. *inaquosorum* (AMXN01000021) |
| P36 (1) | *Stenotrophomonas* (P36) | MN062954 | 95.17 | *Stenotrophomonas maltophilia* (JALV01000036) |
| P37 (4) | *Bacillus subtilis* ssp. *inaquosorum* (P37) | MN062955 | 99.93 | *Bacillus subtilis* ssp. *inaquosorum* (AMXN01000021) |
| P38 (5) | *Bacillus velezensis* (P38) | MN062956 | 99.36 | *Bacillus velezensis* (AY603658) |
| P39 (2) | *Bacillus subtilis* ssp. *inaquosorum* (P39) | MN062957 | 99.3 | *Bacillus subtilis* ssp. *inaquosorum* (AMXN01000021) |
| P40 (1) | *Bacillus paramycoides* (P40) | MN062958 | 99.66 | *Bacillus paramycoides* (MAOI01000012) |
| P41 (1) | *Pseudomonas* (P41) | MN062959 | 98.45 | *Pseudomonas geniculata* (AB021404) |
